# Timing of hospital admission for stillbirth delivery on maternal and obstetric outcome: a retrospective cohort study

**DOI:** 10.1038/s41598-021-98229-9

**Published:** 2021-09-22

**Authors:** Dana Anaïs Muin, Anke Scharrer, Alex Farr, Herbert Kiss, Helmuth Haslacher

**Affiliations:** 1 Division of Feto-Maternal Medicine, Department of Obstetrics and Gynecology, Medical University of Vienna, Waehringer Guertel 18-20, 1090 Vienna, Austria; 2 Clinical Institute for Pathology, Medical University of Vienna, Vienna, Austria; 3 Department of Laboratory Medicine, Medical University of Vienna, Vienna, Austria

**Keywords:** Reproductive signs and symptoms, Physical examination

## Abstract

The effect of timing of hospital admission for stillbirth delivery following late intrauterine fetal death (IUFD) has not yet been described. By this study, we aimed to gain an understanding of the impact of *“immediate”* (i.e., on the same day of IUFD diagnosis) versus *“delayed”* hospital admission (i.e., on the subsequent day or two days after IUFD diagnosis) on maternal and delivery outcome parameters. This retrospective cohort study comprised all women who suffered a singleton IUFD ≥ 21 gestational weeks and delivered the stillborn at our tertiary referral center between 2003 and 2019. We excluded all terminations of pregnancy and women presenting with acute symptoms on the day of IUFD diagnosis. In total, 183 women were included of whom 69.4% (n = 127) were immediately admitted and 30.6% (n = 56) had delayed admission. Median gestational age of IUFD was 30^+3^ (21^+0^–41^+3^) weeks. Whilst women with early signs of labor were more frequently admitted immediately (87.5%; 14/16), neither maternal demographic and obstetric parameters, nor day of the week or presenting symptoms influenced the timing of hospital admission. 77.6% (142/183) of women after IUFD diagnosis delivered within the first 3 days after admission. Women after immediate admission equally often delivered on admission day and the day after (26.0%; 33/127 each), women after delayed admission most commonly delivered the day after admission (39.3%; 22/56). Stillbirth delivery on the day of diagnosis was more common upon immediate admission (*p* = 0.006), especially in early gestational weeks (*p* = 0.003) and with small fetal weight (*p* < 0.001), requiring less induction of labor. No significant difference regarding delivery mode, labor duration, use of intrapartum analgesia, need for episiotomy and risk of perineal injury was observed between the groups. Also rate of intrapartum hemorrhage was independent of admission timing, although immediately admitted women experienced greater median blood loss after vaginal delivery. Maternal laboratory parameters (hemoglobin, thrombocytes and CRP) were independent of admission timing, except for higher levels of leucocytes, neutrophils and lymphocytes in immediately admitted women. Our study shows no clinical superiority of immediate hospital admission for stillbirth delivery. Under stable medical circumstances, it, therefore, seems feasible to allow the woman delayed admission for labor and delivery.

## Introduction

Late intrauterine fetal death (IUFD) occurs with a prevalence of 2.68 per 1000 live births in Austria^1^. The diagnosis is usually made by real-time ultrasonography upon absent fetal heartbeats and umbilical blood flow^2^. Learning the diagnosis of fetal death is immensely distressing and devastating to the affected woman^3,4^. Unless in case of an acute event, such as uterine rupture or placental abruption, current practice guidelines advise that the woman can be discharged home and readmitted at a later stage for induction of labor (IOL), if she remains clinically stable and well^2,5^.

In a recent national survey, we found that 41.3% of Austrian maternity units let the woman decide upon the timing of admission for IOL after fetal death, whereas 17.4% institutions usually admit the woman straight away^6^. The remaining maternity units usually admit the woman on the following day or later. Institutions, which admit the woman immediately, were more likely to perform maternal tests to rule out bleeding and clotting disorders. It has been empirically thought that prolonged uterine retention of the demised fetus increased the maternal risk for disseminated intravascular coagulopathy (DIC) due to toxic releases during the maceration process^7–9^. In this respect, we previously found no association between higher fetal maceration grade and increased risk for maternal DIC^10^.

The paucity of data on the timing of hospital admission for labor induction following IUFD and obstetric outcome, prompted us to design this study to better understand its impact on maternal care and safety. The lack of a clinical standard on the timing of admission following IUFD allowed us to critically evaluate the outcome after immediate versus delayed hospital admission for stillbirth delivery, whilst we hypothesized that the latter was associated with greater maternal and obstetric complications.

## Methods

### Study design and subjects

**Figure 1:**
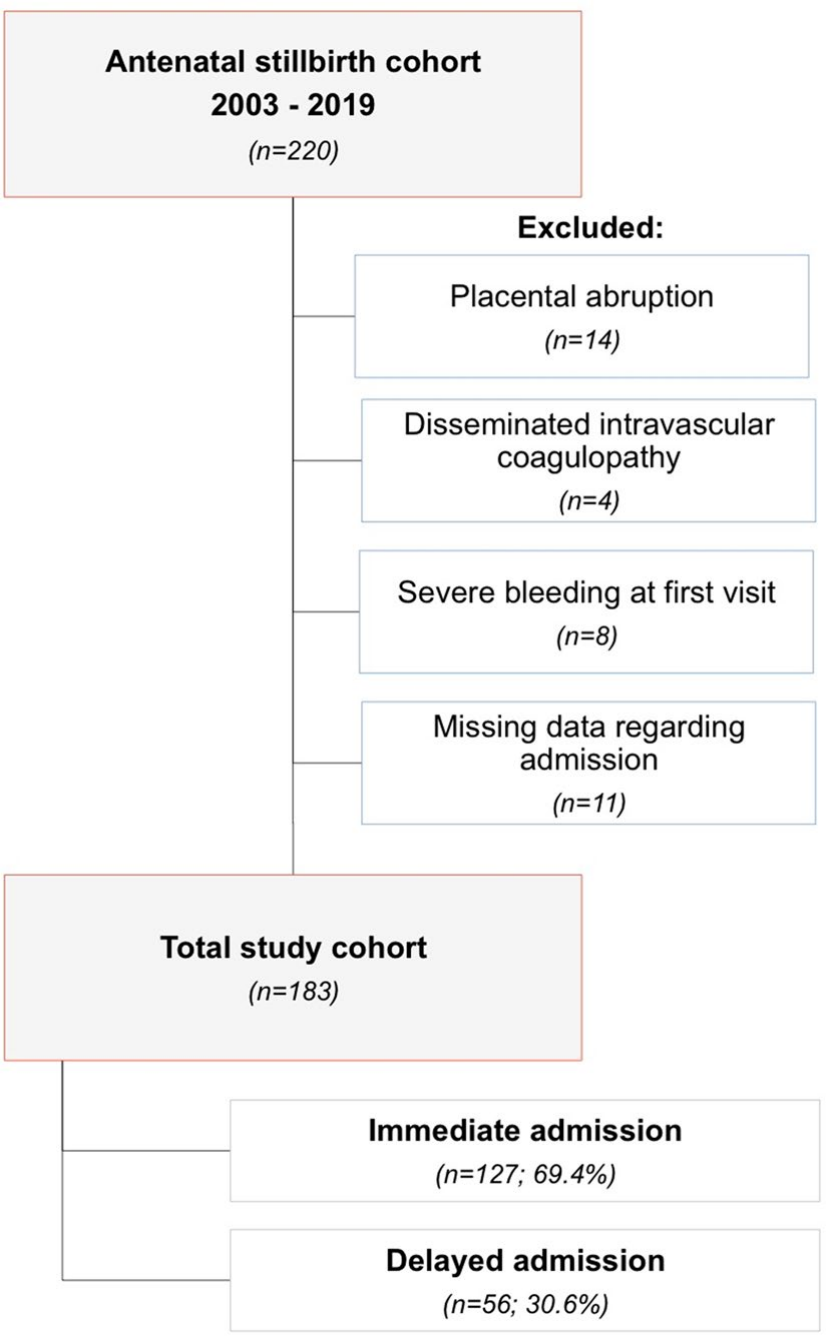
Flowchart on the selection of the study population following fetal death at the Department of Obstetrics and Gynecology, Medical University of Vienna, Austria, between 2003 and 2019.

We conducted a retrospective cohort study on women who suffered singleton IUFD ≥ 21^+0^weeks of gestation and subsequently delivered the stillborn between January 2003 and December 2019 at the Department of Obstetrics and Gynecology at the Medical University of Vienna, Austria. Women were excluded in case of termination of pregnancy, and if they had presented to our department with acute symptoms needing immediate admission and prompt delivery (e.g., acute bleeding, signs of placental abruption, uterine rupture, eclampsia, coagulation disorders, severe maternal or fetal trauma). Cases with missing data regarding the timing of admission were excluded (Fig. 1).

### Data collection

Perinatal, maternal and fetal data were retrieved from the electronic medical database ViewPoint Version 5.6.28.56 (General Electric Company, Solingen, Germany). Variables regarding presenting symptoms, date of diagnosis and date of admission were manually retrieved from the electronic medical records for each case.

Maternal laboratory test results were extracted from the Laboratory Information and Management System MOLIS (vision4health, Bochum, Germany) at the Department of Laboratory Medicine at the Medical University of Vienna, Austria.

Upon admission, all women after IUFD routinely received laboratory tests including assessment of their complete blood count [neutrophils, lymphocytes (Lymphos), monocytes (Monos), eosinophils (Eos), basophils (Baso), erythrocytes (Ery), hemoglobin (Hb), leucocytes (Lc), thrombocytes (Tc)], coagulation parameters and C-reactive protein (CRP).

Biochemical analyses were conducted at the Department of Laboratory Medicine, Medical University of Vienna, Austria (ISO9001-certification; ISO15189-accreditation). Complete blood count and CRP were quantified on Sysmex hematology analyzers (Sysmex Europe GmbH, Norderstedt, Germany). Results below the limit of detection (LOD) were estimated by their assumed expected value: $\overline{x }=\frac{0+LOD}{2}$ and values above the highest point of the standard curve (HQS) were fixed at ${x}_{h}=HQS+1$. At our institution, normal ranges are as follows: Lc 4.0–10.0 G/L, CRP < 0.5 mg/dl, Hb 12.0–16.0 g/dl, Tc 150–350 G/L, Neutrophils (absolute) 2.0–7.5 G/L, Neutrophils (relative) 50.0–75.0%, Lymphos (relative) 25.0–40.0%, Monos (relative) 0.0–12.0%.

All data were transferred into a database, checked for accuracy and consistency and made anonymous prior to analyses.

### Definitions

At our institution, clinically stable women are offered to freely decide on the timing of their hospital admission after stillbirth. For this retrospective study, women were included into the *“immediate admission”* group, if they had decided to be admitted to the hospital on the same day of IUFD diagnosis. Women of “*delayed admission*" had opted for admission on the subsequent day or 2 days after the diagnosis of fetal death.

Symptoms at diagnosis were grouped into *fetal* (reduced fetal movements), *maternal* (bleeding, cardiovascular symptoms, discomfort/pain, pain accompanied by bleeding), *early signs of labor* (bulging membranes, rupture of membranes, contractions) and *none* (e.g., a woman attending routine antenatal check-up).

Maternal age was defined as age in years at the time of delivery. Ethnicity was self-reported by the woman. Gravidity was defined as the number of the current pregnancy. Parity was defined as the number of previous deliveries. Maternal weight (in kg) was obtained at antenatal booking or admission and Body-Mass-Index (BMI) was grouped into six categories: underweight (≤ 18.5 kg/m^2^), normal weight (18.6–24.9 kg/m^2^), pre-obesity (25–29.9 kg/m^2^), obesity class I (30–34.9 kg/m^2^), obesity class II (35–39.9 kg/m^2^) and obesity class III (≥ 40 kg/m^2^). Smoking was defined as current smoker or non-smoker during pregnancy.

Indications for lower segment caesarean sections (LSCS) were categorized according to the Robson classification based on parity, previous LSCS, LSCS before labor or after failed IOL^11^.

Intrapartum obstetric hemorrhage was defined as an estimated blood loss ≥ 500 ml during vaginal birth or ≥ 1000 ml during LSCS^12^.

As per Austrian law, each fetus undergoes a standard post-mortem examination including autopsy (and occasionally fetal imaging). Fetal autopsies were conducted in all cases according to standard operating procedures at the Clinical Institute for Pathology, Medical University of Vienna, Austria. Upon receipt of all post-mortem reports (including genetic testing, placental histology, and maternal tests results), we followed the algorithm as proposed by Reddy et al. to conclude the cause of fetal death^13^. Cause of fetal death was defined as the “initial, demonstrable pathophysiological entity initiating the chain of events that has irreversibly led to death” and categorized according to the Tulip classification^14^.

Fetal maceration was graded by the perinatal pathologist according to international standards (*grade 0:* “parboiled” skin discoloration; *grade I:* not-specified desquamation of the skin; *grade II:* bloodstained effusions in cavities and skin peeling; *grade III:* yellow–brown liver, turbid effusions and mummification)^8^.

### Statistical analysis

The distribution of data was analyzed using the Kolmogorov–Smirnov test. Normally distributed continuous data are expressed as mean ± standard deviation. Not normally distributed variables are expressed as median and minimum–maximum. Categorical data are presented as frequencies (*n*) and proportions (*%*). Continuous data were compared with unpaired *t*-test and Mann–Whitney *U* test, respectively. Categorical data were compared with Chi^2^ and Fisher’s Exact test, respectively, with a 99% Confidence Interval (CI). Spearman’s rank correlation coefficient was used to compare ordinal and continuous variables. Binary logistic regression analysis was done to calculate the odds ratios (OR) and a 95% CI for the relationship of categorical variables. A forest plot with a logarithmic scale (log 10) illustrates the OR and 95% CIs. All reported *p*-values are two-sided, and the level of significance was set at < 0.05. Statistical tests were performed with SPSS Statistics Version 26.0.0.0 (IBM Corporation, Armonk, NY, USA) and figures created by GraphPad Prism 9 for macOS Version 10.14.6 (GraphPad Software, LLC).

### Ethical approval

The study was approved by the Ethics Committee of the Medical University of Vienna (*Registration number 1202/2018*) and complied with the principles outlined in the Declaration of Helsinki of 1975, as revised in 2013. Participants’ written consent was not required per the Austrian Federal Act concerning Protection of Personal Data (DSG 2000). All patient data were de-identified before analyses.

## Results

### Maternal baseline characteristics

In total, we included 183 women whose baseline characteristics are shown in Table 1**.** In our study cohort, fetal death was most frequently diagnosed during routine antenatal checks (56.3%; 103/183), upon reduced fetal movements (27.9%; 51/183), due to suspected contractions (6.0%; 11/183), lower abdominal discomfort (3.3%; 6/183), rupture of membranes (2.7%; 5/183), vaginal spotting (1.1%; 2/183), maternal cardiovascular symptoms (1.1%; 2/183), pain with light bleeding (1.1%; 2/183), and bulging membranes (0.5%; 1/183). In one case (0.5%), symptoms were not reported on the day of diagnosis.

**Table 1:** Study sample characteristics (n = 183) after hospital admission for stillbirth delivery at the Department of Obstetrics and Gynecology, Medical University of Vienna between 2003 and 2019.

Maternal characteristics		n	%
Age at stillbirth (years)^a^		31 ± 7	
Ethnicity	Middle European	86	47
	Eastern European	48	26.2
	Turkish	26	14.2
	Middle Eastern	10	5.5
	African	7	3.8
	South Asian, Indian	3	1.6
	American	2	1.1
	Chinese	1	0.5
Gravida (*n*)^b^		2 (1–12)	
Para (*n*)^b^		1 (0–9)	
Body mass index (kg/m^2^)^b^		24.5 (14.6–43.3)	
BMI categories	Underweight	4	2.9
	Normal	71	51.1
	Pre-obese	44	31.7
	Obesity class I	9	6.5
	Obesity class II	8	5.8
	Obesity class III	3	2.2
Nicotine consumption		36	23.5
Hypertensive disorders in pregnancy	Pre-existing hypertension	12	6.6
	Pre-eclampsia	14	7.7
	Pregnancy induced hypertension	7	3.8
	HELLP	4	2.2
Diabetes	Insulin-dependent gestational diabetes	17	9.3
	Pre-existing diabetes	4	2.2
	Gestational diabetes (diet only)	3	1.6
Co-morbidities	Other medical	35	19.1
	Hemostatic	15	8.2
	Auto-immune disorder	11	6
	Antiphospholipid Syndrome	6	3.3
	Gynecological	5	2.7
	Cardiac	3	1.6
	Neurological	2	1.1
	Mental	2	1.1
	Respiratory	0	0
Total number of co-morbidities (*n*)	0	105	57.4
	1	61	33.3
	2	13	7.1
	3	2	1.1
	4	2	1.1
Social restriction/domestic violence		5	2.7

*BMI* body mass index, *HELLP* hemolysis, elevated liver enzymes, low platelets.

^a^Mean ± standard deviation.

^b^Median (minimum – maximum).

### Fetal baseline characteristics

The IUFD cohort consisted of 51.4% (94/183) male and 48.6% (89/183) female fetuses between 21^+0^ and 41^+3^ gestational weeks with a median age of 30^+3^ gestational weeks. Median fetal weight at stillbirth was 1020 (180–4450) g.

Abnormal fetal post-mortem findings and/or prenatally confirmed congenital malformations were reported in 54.6% (100/183) cases. Causes of fetal death were confined as placental pathologies in 19.1% (35/183) cases; unknown due to lack of important information in 13.1% (24/183) cases; maternal or fetal disease in 6.6% (12/183) cases; unknown despite thorough investigation in 4.9% (9/183) cases and infection in 1.6% (3/183) cases.

Among the fetuses with post-mortem anomalies, 49.0% (49/100) had involvement of two or more organ systems. 22.0% (22/100) fetuses had anomalies of the heart and circulatory system; 12.0% (12/100) had chromosomal aberrations; 6.0% (6/100) had malformations of the musculoskeletal system and 4.0% (4/100) fetuses had anomalies in the respiratory and digestive system, respectively. In 3.0% (3/100) fetuses we detected a microdeletion defect or anomaly of the central nervous system or urogenital system.

### Factors influencing the timing of admission

In our cohort, women most commonly presented on Mondays (21.9%; 37/183), Thursdays and Fridays (17.8% each; 30/183; Supplementary Fig. 1) as the IUFD was diagnosed. Excluding all women who presented with acute symptoms requiring rapid admission and immediate delivery (e.g., established labor, placental abruption, uterine rupture), at our institution, 69.4% (127/183) women were admitted on the same day of IUFD diagnosis, and 30.6% (56/183) women chose to delay their admission (Table 2).

**Table 2:** Comparison of fetomaternal characteristics and obstetric variables after stillbirth delivery between immediate and delayed admission groups.

			Total (n = 183)	Immediate admission (n = 127)	Delayed admission (n = 56)	*p-*value
Maternal	Maternal age (years)^a^		31 ± 7	31 ± 6	32 ± 7	0.094^d^
	Body mass index (kg/m^2^)^b^		24.5 (15.6–43.3)	25.1 (15.6–43.3)	24.2 (18.6–37.6)	0.669^e^
	Para (*n*)^b^		1 (0–9)	0 (0–9)	1 (0–9)	0.529^e^
	Previous LSCS (*n*)	None	144 (78.7%)	99 (78.0%)	45 (80.4%)	0.847^f^
		1	30 (16.4%)	21 (16.5%)	9 (16.1%)	
		2	9 (4.9%)	7 (5.5%)	2 (3.6%)	
	Presenting symptoms	None	103 (56.6%)	71 (56.3%)	32 (57.1%)	0.473^f^
		Fetal	51 (28.0%)	31 (24.6%)	20 (35.7%)	
		Maternal	12 (6.6%)	10 (7.9%)	2 (3.6%)	
		Labor	16 (8.8%)	14 (11.1%)	2 (3.6%)	
		Not reported	0 (0.0%)	1 (0.1%)	0 (0.0%)	
Fetal	Gestational age (days)^b^		213 (147–290)	209 (147–290)	221 (152–288)	0.331^e^
	Maceration grade	0	17 (11.6%)	16 (15.1%)	1 (2.5%)	< 0.001^f^
		I	20 (13.7%)	17 (16.0%)	3 (7.5%)	
		II	43 (29.5%)	34 (32.1%)	9 (22.5%)	
		III	66 (45.2%)	39 (36.8%)	27 (67.5%)	
Obstetrics	Delivery mode^c^	Vaginal cephalic	110 (60.1%)	78 (61.4%)	32 (57.1%)	0.465^f^
		Vaginal breech	53 (29.0%)	35 (27.6%)	18 (32.1%)	
		Ventouse	3 (1.6%)	1 (0.8%)	2 (3.6%)	
		LSCS	17 (9.3%)	13 (10.3%)	4 (7.1%)	
	Robson classification of LSCS	10C	8 (47.1%)	7 (53.8%)	1 (25%)	0.130^f^
		10B	2 (11.8%)	2 (15.4%)	0 (0.0%)	
		5C	2 (11.8%)	2 (15.4%)	0 (0.0%)	
		6C	2 (11.8%)	1 (7.7%)	1 (25.0%)	
		4B	1 (5.9%)	0 (0.0%)	1 (25.0%)	
		6B	1 (5.9%)	1 (7.7%)	0 (0.0%)	
		7C	1 (5.9%)	0 (0.0%)	1 (25.0%)	
	Time length of vaginal delivery (h)^b^		3 (0–28)	3 (0–28)	4 (0–26)	0.231^e^
	2nd stage of labor (min)^b^		10 (0–376)	10 (0–289)	18 (0–376)	0.197^e^
	3rd stage of labor (min)^b^		10 (0–90)	10 (0–78)	10 (0–90)	0.323^e^
	Blood loss (ml)^b^		200 (30–2500)	200 (30–2500)	150 (50–1000)	0.064^e^
	Anesthesia^c^	None	100 (54.6%)	63 (49.6%)	37 (66.1%)	0.089^f^
		Peridural	36 (19.7%)	26 (20.5%)	10 (17.9%)	
		Epidural	21 (11.5%)	19 (20.5%)	2 (3.6%)	
		Spinal	14 (7.7%)	10 (7.9%)	4 (7.1%)	
		Intubation	11 (6.0%)	9 (7.1%)	2 (3.6%)	
		Local	1 (0.5%)	0 (0.0%)	1 (1.8%)	
	Episiotomy^c^	None	181 (98.9%)	126 (99.2%)	55 (98.2%)	0.549^f^
		Mediolateral	2 (1.1%)	1 (0.8%)	1 (1.8%)	
	Perineal injury^c^	None	154 (84.2%)	107 (84.3%)	47 (83.9%)	0.358^f^
		Vaginal tear	9 (4.9%)	8 (6.3%)	1 (1.8%)	
		1st degree tear	7 (3.8%)	5 (3.9%)	2 (3.6%)	
		Labial tear	6 (3.3%)	2 (1.6%)	4 (7.1%)	
		2nd degree tear	4 (2.2%)	3 (2.4%)	1 (1.8%)	
		Cervical tear	3 (1.6%)	2 (1.6%)	1 (1.8%)	

*LSCS* lower segment caesarean section.

^a^Mean ± Standard deviation.

^b^Median (minimum − maximum).

^c^Frequency (proportion).

^d^Unpaired t-test with level of significance < 0.05.

^e^Mann–Whitney U test with level of significance < 0.05.

^f^Fishers’s exact test with level of significance < 0.05.

Robson classification: 10C: All singleton cephalic, ≤ 36 weeks (including previous LSCS): LSCS before labor; 10B: All singleton cephalic, ≤ 36 weeks (including previous LSCS): induced labor; 5C: Previous LSCS, singleton cephalic, ≥ 37 weeks: LSCS before labor; 6C: All nulliparous breeches: LSCS before labor; 4B: Multipara, singleton cephalic, ≥ 37 weeks: induced labor; 6B: All nulliparous breeches: induced labor; 7C: All multiparous breeches (including previous LSCS): LSCS before labor.

**Figure 2:**
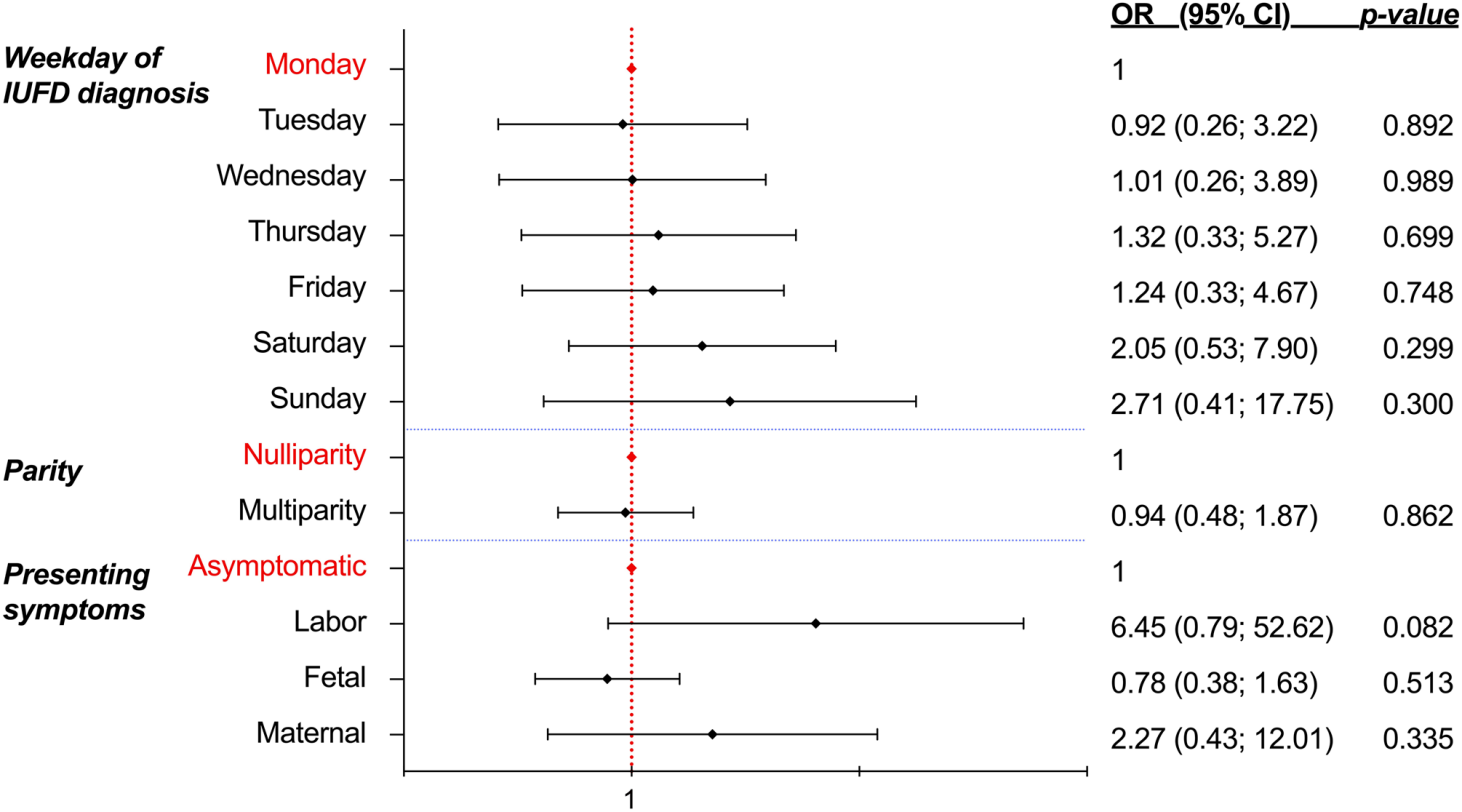
Forest plot illustrating the odds ratios (and 95% Confidence Intervals) from multivariate logistic regression to identify the relationship between timing of hospital admission and possible predictors (i.e., weekday, parity, presenting symptom at diagnosis) following intrauterine fetal death (n = 183; level of significance p < 0.05, two-tailed). *IUFD* intrauterine fetal death, *OR* odds ratio, *CI* confidence interval. Figure software: Prism 9 for macOS, Version 9.2.0.

Whilst women presenting with early signs of labor (8.7%; 16/183), such as bulging membranes, rupture of membranes or sporadic contractions, were more frequently admitted immediately (87.5%; 14/16), there was no statistically significant likelihood of being admitted immediately regarding presenting symptoms at diagnosis (Fig. 2). Likewise, neither specific day of the week, nor multiparity were related with timing of hospital admission (*p* = 0.694 and *p* = 0.862; Fig. 2).

### Influence on delivery time

**Figure 3:**
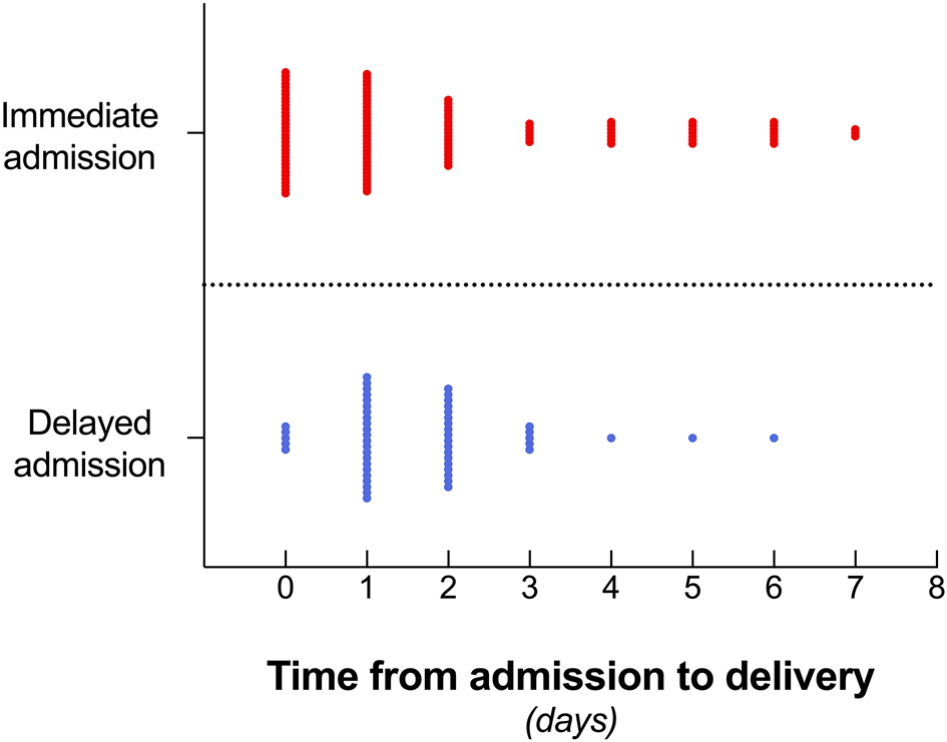
Time to stillbirth delivery after immediate (n = 127) and delayed (n = 56) hospital admission (nested scatter dot plot; width of distribution of points proportional to the number of delivered women; Day 0 = hospital admission day). Stillbirth delivery occurred in 20.8% (38/183) on *Day 0*; in 30.1% (55/183) on *Day 1*, and in 20.2% (37/183) on *Day 3*. Figure software: Prism 9 for macOS, Version 9.2.0.

The time interval from admission to stillbirth delivery is illustrated in Fig. 3, highlighting the trend for delivery within the first three days in 77.6% (142/183) of women after IUFD diagnosis.

On admission day (i.e., *Day 0),* 26.0% (33/127) women gave birth after immediate admission, whilst only 8.9% (5/56) women did after delayed admission (*p* = 0.006).

**Figure 4:**
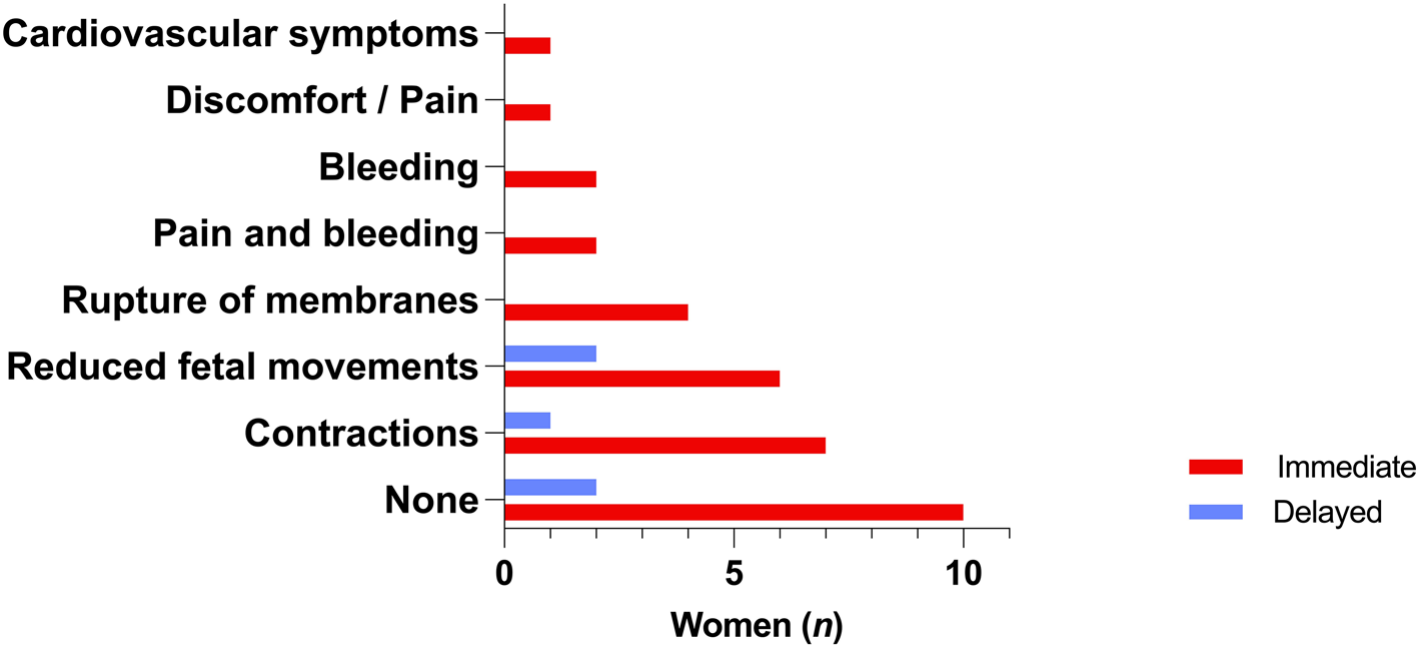
Presenting symptoms on day of IUFD diagnosis in women who delivered on the same day of hospitalization (n = 33 immediate admission; n = 5 delayed admission). Figure software: Prism 9 for macOS, Version 9.2.0.

Immediate admission and same-day-delivery significantly correlated with early gestational weeks (Spearman’s *r* = −0.287; *p* = 0.003) and small fetal weight (*r* = −0.337; *p* < 0.001), yet not with parity (*p* = 0.06). The majority of women after immediate admission and same-day-delivery had equally common presented with early signs of labor (33.3%; 11/33) and no symptoms at all (30.3%; 10/33). The rest had experienced reduced fetal movements (18.2%; 6/33), or maternal symptoms (18.2%; 6/33; Fig. 4).

Delivery on the first day after admission (i.e., *Day 1)* occurred in 26.0% (33/127) women after immediate versus 39.3% (22/56) women after delayed admission (*p* = 0.382).

Delivery on the second day after admission (i.e., *Day 2)* occurred in 15.0% (19/127) women after immediate admission versus 32.1% (18/56) women after delayed admission (*p* = 0.006). Likelihood for delivery from days 3 to 6 was equal in both groups.

Naturally, fetal maceration grade was lower after immediate than delayed admission (grade 0 versus grade III; *p* < 0.001; Table 2).

### Methods for induction of labor

**Figure 5:**
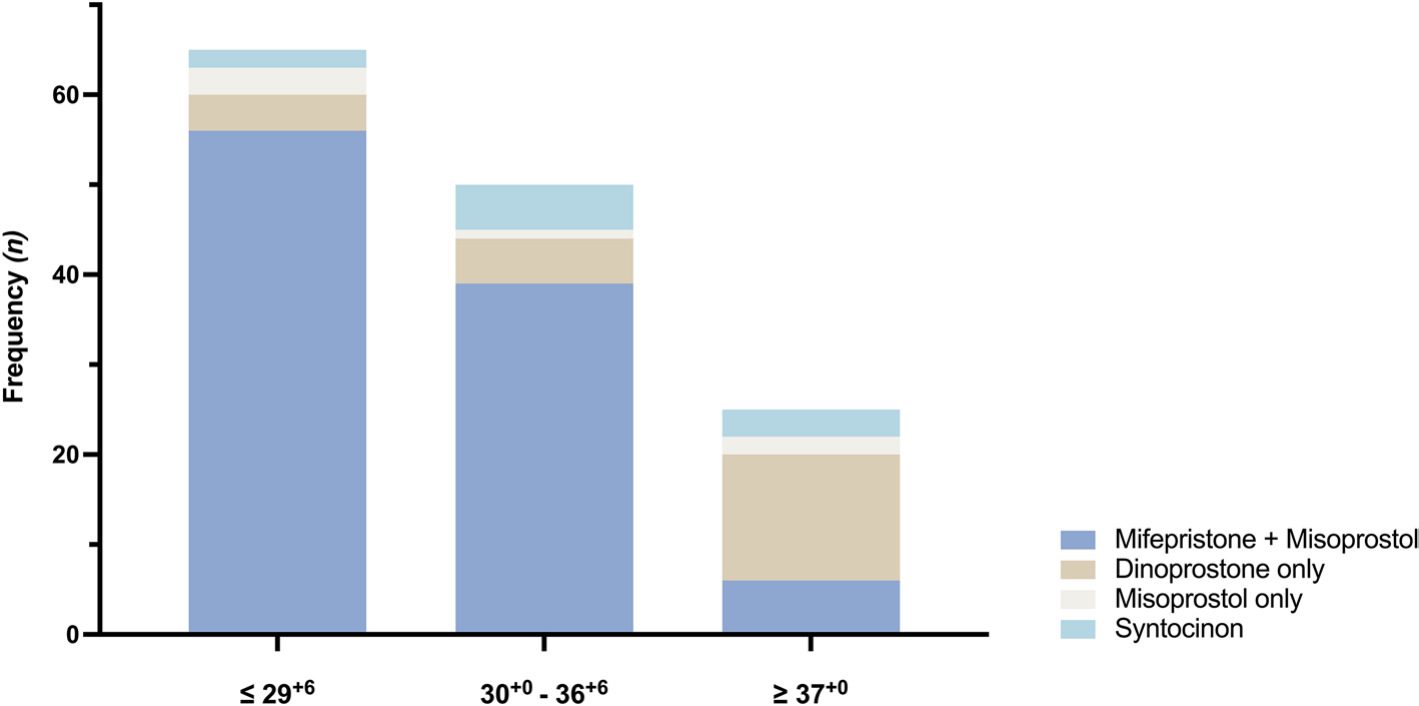
Methods of induction of labor following fetal death at the Department of Obstetrics and Gynecology, Medical University of Vienna, Austria, between 2003 and 2019 (“*Dinoprostone only”* includes Propess, Prostin E2; *“Misoprostol only”* includes Cytotec, Misodel, Cyprostol). Figure software: Prism 9 for macOS, Version 9.2.0.

In the total study cohort, most common method for IOL was mifepristone (on *Day 0*) with misoprostol (on *Day 1*) in 55.2% (101/183) of the cases, followed by dinoprostone only (12.6%; 23/183), misoprostol only (3.3%; 6/183) and intravenous syntocinon (5.5%; 10/183). Labor induction failed or was omitted in 23.5% (43/183) of women (Fig. 5).

Women, who were admitted immediately, required less IOL [18.9% (2/127) vs. 1.7% (1/56); *p* = 0.001], whilst women after delayed admission were more frequently induced with mifepristone combined with misoprostol [73.2% (41/56) vs. 47.2% (60/127); *p* = 0.002].

### Labor and delivery

Methods of delivery and indications for LSCS were independent of timing of admission (Table 2). In our cohort, 9.3% (17/183) women were delivered by LSCS, of whom 41.2% (7/17) women had at least one previous LSCS. Indications for LSCS were maternal co-morbidities requiring primary LSCS (35.3%; 6/17), maternal request (29.4%; 5/17), failed IOL (17.6%; 3/17), two previous LSCS (11.8%; 2/17) and transverse fetal lie in anhydramnios (5.9%; 1/17).

### Obstetric outcome

In total, intrapartum hemorrhage was observed in 10.7% (6/56) of delayed and 17.3% (22/127) of immediately admitted women, however, no significant odds were described in women following immediate hospital admission [OR 1.74 (95% CI 0.66–4.57); *p* = 0.257]. Total amount of blood loss was independent of admission timing (Table 2), however, during cephalic vaginal delivery, immediately admitted women experienced greater median blood loss [200 ml (30–2500 ml) vs. 150 ml (50–600 ml), *p* = 0.041; Supplementary Fig. 2].

Yet, again, labor time, need for analgesia, episiotomy and prevalence of perineal injury were independent of admission timing (Table 2).

### Maternal laboratory parameters

**Figure 6:**
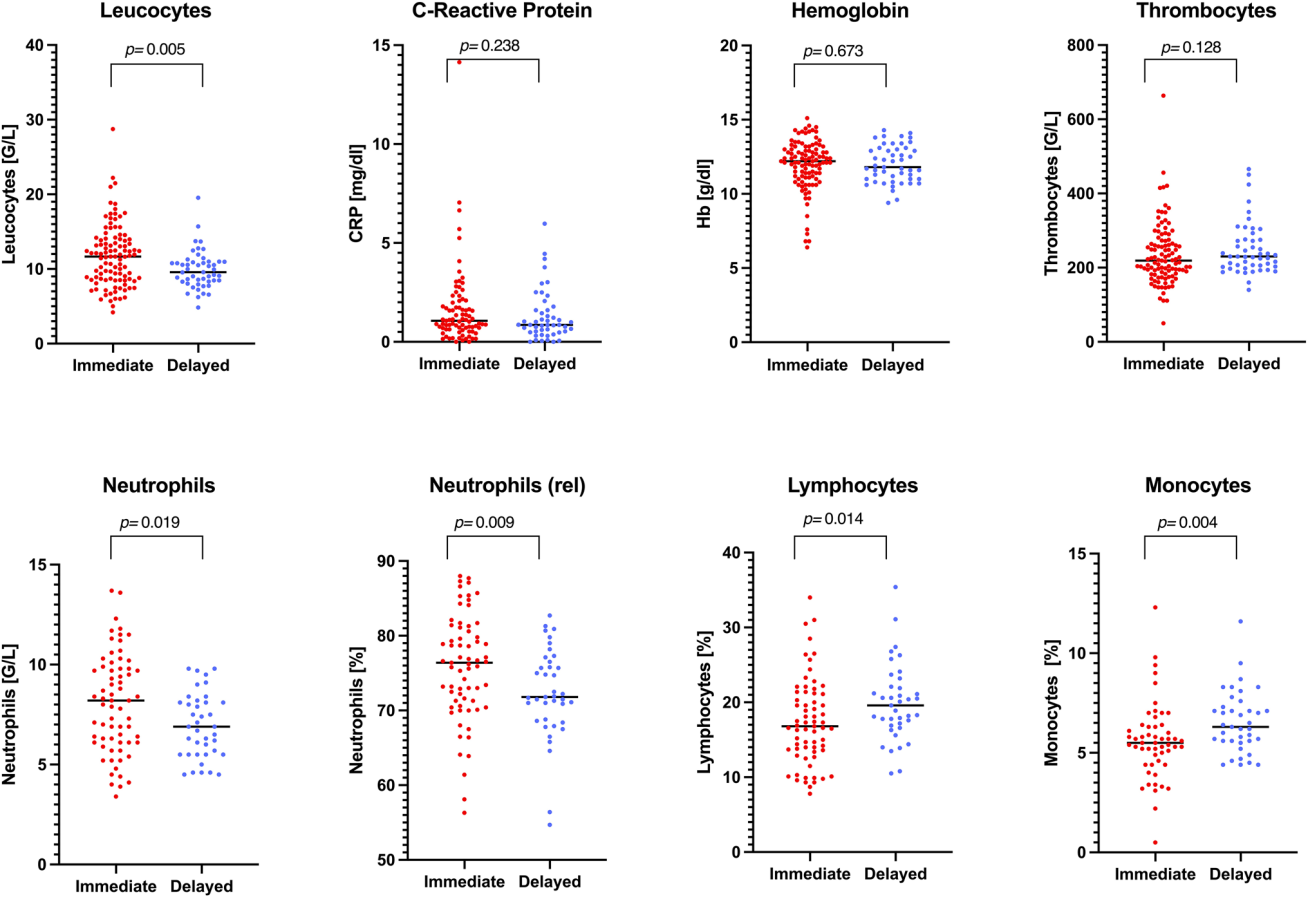
Comparison of maternal laboratory values between immediate versus delayed admission (n = 166, excluding all women who delivered by caesarean section; Scatter dot plot with line at median; Mann–Whitney *U* test; two-tailed Exact *p-*value with 99% confidence interval). Figure software: Prism 9 for macOS, Version 9.2.0.

Irrespective of presenting symptoms at diagnosis, upon immediate admission, women had higher levels of leucocytes (*p* = 0.006), absolute and relative neutrophils (*p* = 0.019 and *p* = 0.009, respectively) and lymphocytes (*p* = 0.014). Delayed admission was associated with higher monocyte counts (*p* = 0.004). Hemoglobin levels, thrombocytes and CRP were similar in all women, excluding those delivered by LSCS (Fig. 6).

## Discussion

It is widely recognized that the diagnosis of fetal death causes great emotional turmoil in the affected parents. It usually takes a certain time to come to terms with the sad diagnosis, and every woman adapts differently to her coping strategies until she feels capable of handling the experience of birth and death of her child colliding, leaving a long-lasting impact on herself and her partner. The admission to hospital for giving birth to the deceased baby is an important event, and the medical care during this time may have a profound effect on the future life of the bereaved family^15^.

By this study, we sought to elaborate on the clinical impact of the timing of hospital admission on maternal and obstetric parameters to gather more evidence regarding care after fetal death during hospitalization. In the small sample of our retrospective study, the affected women themselves selected the timing of their hospital admission, unless they presented with immediate signs of labor or as an emergency. The majority of cases of fetal death in this cohort were diagnosed during routine controls, and more than half of the women were admitted on the same day of diagnosis. Upon admission, there was no prevalence in presenting symptoms, yet women with early signs of labor chose to be admitted immediately. Stillbirth delivery most commonly took place on the first day after admission, yet immediately admitted women equally often delivered on admission day and the day after, while delayed admitted women more commonly delivered on the day after admission, often following IOL. Women, who were admitted immediately after diagnosis of fetal death, yet also delivered on the day of their admission, were either in early gestational weeks and/or bared smaller fetal weight. Also, we observed a reactive left-shift in the maternal laboratory parameters upon immediate admission: We assume that this might have either contributed to accelerated laboring and delivery, or may have reflected the inflammatory response of labor itself. Finally, in our cohort, about one in ten women required LSCS following fetal death, the indications of which were maternal comorbidities in the majority of cases, maternal request, or failed IOL. Whilst the rate of intrapartum hemorrhage showed no group prevalence, generally, immediately admitted women had a greater median amount of blood loss after vaginal delivery.

Despite the lack of comparable studies against which we may interpret our findings, our retrospective analysis shows, that from a clinical standpoint, there seems to be no harm in allowing a delayed hospital admission for induction following diagnosis of fetal death, neither in labor time, analgesia, birth injuries, and total blood loss, nor in clinically relevant laboratory parameters, such as maternal hemoglobin, thrombocytes and CRP. These findings underpin our previous observations, that maternal clotting disorders are uncommon and rarely directly related to fetal death as such^10^.

Whilst our study is the first to provide data on the impact of admission timing in a comprehensively investigated cohort of singleton IUFDs, we acknowledge several study limitations inherent to its retrospective design and single-center nature: First and foremost, the small sample size may limit the power and significance of the results. The sampling of the subgroups (e.g., mode of delivery; symptoms at diagnosis, methods for IOL) might have further diminished the strength of the analyses in the compared groups. Also, the length of “delay” of hospital admission was restricted to one to two days only, therefore, our results may not be generalizable and translatable into other settings, where a longer watchful waiting or active surveillance is practiced after IUFD. We, furthermore, acknowledge the failure to control for a possible selection bias in admission timing, either patient or caregiver induced, and the different lengths of maternal exposure to the deceased fetus in utero, which may have triggered labor, omitting the need for IOL, yet accelerating admission. Finally, we could not adjust for errors and inconsistency in the medical documentation resulting in a recall bias by the attending physician at the time of IUFD diagnosis and hospital admission.

After all, these limitations indicate that deeper research is needed. We propose, that in the ViewPoint software, the electronic establishment of a structured under-category for the rare event of IUFD documentation (with tick-boxes) would be of great value for future retrospective studies allowing direct and precise retrieval of a set of important parameters, such as (a) *the main circumstance that led to consultation and subsequent diagnosis*; (b) *last time when the baby was felt*; (c) *any precipitating symptoms*; (d) *any recent illness or deterioration of a specific co-morbidity which might have contributed to this event*; (e) *woman’s preference regarding the time of admission/method of delivery*. Also, qualitative evaluation of the motivation for immediate versus delayed admission may provide important insights to improve care for the affected women.

## Conclusion

Our study shows no clinical superiority of immediate over delayed hospital admission for stillbirth delivery. Under stable medical circumstances, it, therefore, seems feasible to allow the woman to be discharged home following the diagnosis of fetal death and be readmitted at a later time point for labor and delivery, when appropriate.

## Supplementary Information

Supplementary Figure S1.

Supplementary Figure S2.
