# Supplementary figures and images for: Timing of hospital admission for stillbirth delivery on maternal and obstetric outcome: a retrospective cohort study
Source: Sci Rep. 2021 Sep 22;11:18854. doi: 10.1038/s41598-021-98229-9 (PMC8458494; doi:10.1038/s41598-021-98229-9)

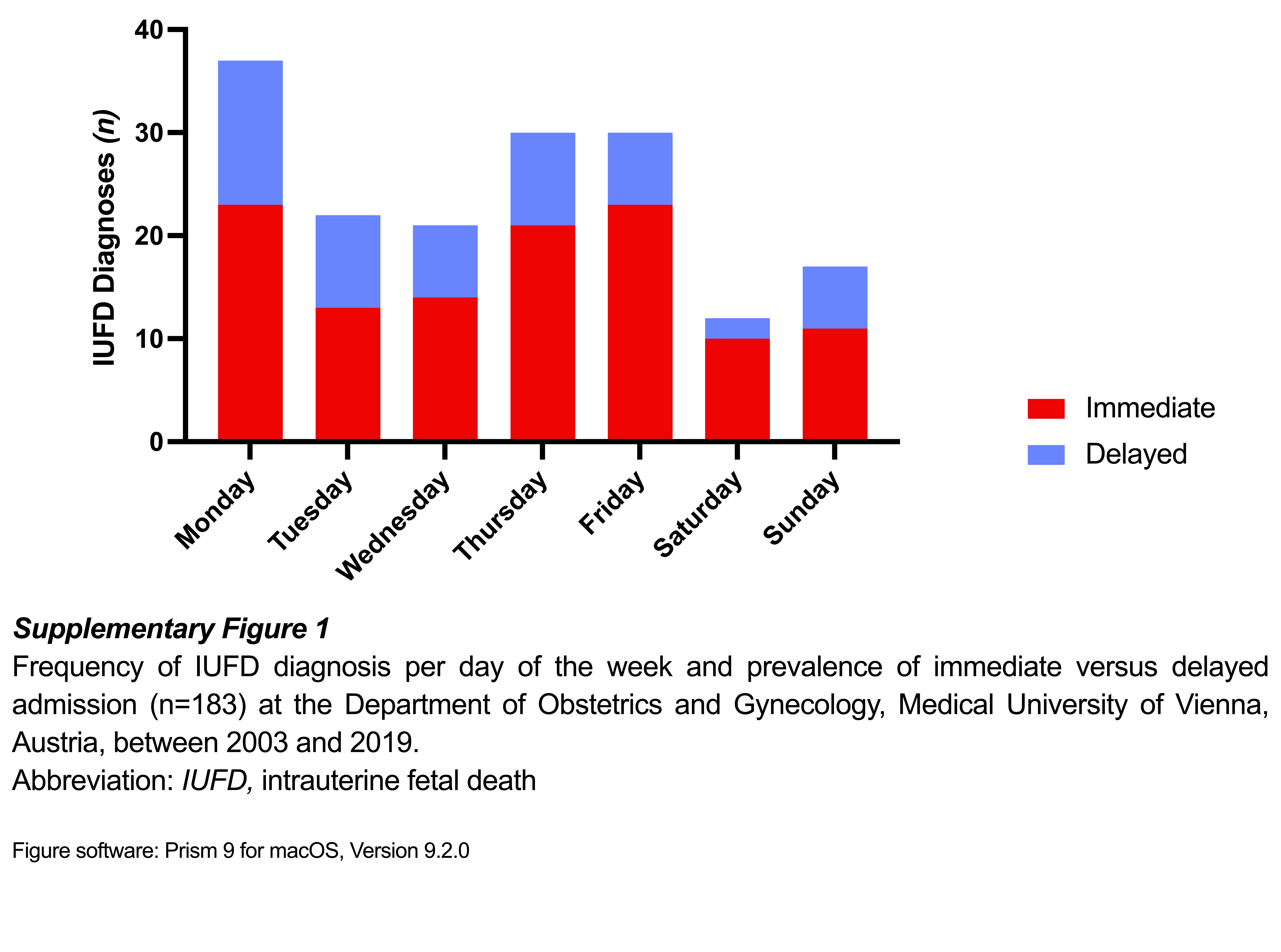

Supplement: Supplementary file 1 — Supplementary Figure S1. [file 41598_2021_98229_MOESM1_ESM.jpg]
